# Phylogenetic, genomic and morphological characterization of Rickettsia senegalensis sp. nov., a novel Rickettsia species detected worldwide

**DOI:** 10.1099/ijsem.0.007294

**Published:** 2026-10-08

**Authors:** Clément Labarrere, Cheikh Tidiane Houmenou, Pierre-Edouard Fournier, Florence Fenollar, Oleg Mediannikov

**Affiliations:** 1 IHU-Méditerranée Infection, Marseille, France; 2 Microbes Evolution Phylogeny and Infections (MEPHI), Aix-Marseille Université, AP-HM, Marseille, France; 3 Infectious Risks in the Tropics and Emerging Microorganisms (RITMES), Aix-Marseille Université, SSA, AP-HM, Marseille, France; 4 CNR des rickettsioses, de la fièvre Q et des bartonelloses, IHU Méditerranée Infection, Marseille, France; 5 IRD, Marseille, France

**Keywords:** *Rickettsia senegalensis*, *Ctenocephalides felis*, fleas, *Rickettsiosis*

## Abstract

Members of the genus *Rickettsia* are widespread obligate intracellular bacteria with high medical and veterinary relevance. While *Rickettsia senegalensis* was previously isolated from cat fleas, *Ctenocephalides felis*, in Senegal, a comprehensive polyphasic characterization is required to validly publish its name. This study aims to provide the first detailed phenotypic, ultrastructural and genomic characterization of the type strain PU01-02ᵀ. Cultivation assays demonstrated that strain PU01-02ᵀ grows aerobically in XTC-2, SF9 and LD652 cell lines at 28 °C in a CO_2_-free atmosphere. Ultrastructural examination via scanning electron microscopy using a backscattered electron detector confirmed its free cytoplasmic localization and a typical rickettsial morphology characterized by rod-shaped cells ~1.5 µm in length and 0.3 µm in width. Whole-genome sequencing yielded a 1.62 Mb genome with a G+C content of 33.2%, and three plasmids were detected. Genomic analyses confirmed its status as a distinct species, placing it within the transitional *Rickettsia* group, specifically inside the *Rickettsia felis* cluster. A review of epidemiological data revealed that rickettsial genes identical to those of *R. senegalensis* had already been identified in several haematophagous arthropods, including fleas and ticks parasitizing various hosts such as cats, dogs, opossums and rodents worldwide. It has also been detected in cat tissues, suggesting a potential host-pathogen association.

Based on these comprehensive characterization data, we formally propose *R. senegalensis* sp. nov. as a new species, with PU01-02ᵀ (=CSUR R184ᵀ =DSM 28250ᵀ) as the type strain.

## Introduction

*Rickettsia* species (*Rickettsiales*, *Rickettsiaceae*) are Gram-negative, obligate intracellular bacteria responsible for various human diseases, including epidemic typhus [1]. To date, the genus *Rickettsia* comprises more than 30 species, 22 of which are pathogenic to humans [2, 3]. These species are classified into four groups based on their genomic, epidemiological and clinical characteristics: the ancestral group, the spotted fever group, the typhus group and the transitional group (TRG) [4, 5].

Several species within the TRG, such as *Rickettsia akari* and *Rickettsia australis*, are well-established human pathogens [6, 7]. More recently, increasing attention has been given to *Rickettsia felis* and closely related species within this group. *R. felis* has been detected in more than 20 different species of haematophagous arthropods worldwide, including fleas, *Anopheles* mosquitoes, lice, soft and hard ticks [8–10]. Non-haematophagous booklice *Liposcelis bostrychophila* were also found to harbour *R. felis* [11]. Considered an emerging *Rickettsia*, *R. felis* has been implicated in cases of fever reported on every continent except Antarctica. Studies have demonstrated that *R. felis* is a significant cause of febrile illnesses of unknown origin, accounting for up to 15% of cases in sub-Saharan Africa and reaching 19.6% in parts of Asia [12–16]. It seems that *R. felis*-associated fever is present in tropical and sub-tropical regions all over the world and sometimes may be quite prevalent. *R. felis* is the most extensively studied species within a cluster that comprises multiple arthropod-associated bacteria, most of which have not yet been isolated and characterized [17, 18]. However, the genetically close *Rickettsia asembonensis* species, recently described from fleas, was also implicated in human pathology [19, 20]. Consequently, characterizing other members of this *R. felis* cluster is critical, as they share similar arthropod vectors and geographic niches, yet their specific medical and veterinary significance remains largely unresolved.

In this context, this study describes *Rickettsia senegalensis*, another prominent member of the *R. felis* cluster. Isolated in 2012 from cat fleas, *Ctenocephalides felis*, collected in Dakar, Senegal, this species has since been detected multiple times in different regions worldwide (Fig. 1) [21]. It has been identified in various haematophagous arthropods, including fleas parasitizing cats, dogs, opossums and rodents (*C. felis, Ctenocephalides orientis, Coptopsylla lamellifer, Xenopsylla skrjabini, Xenopsylla gerbilli* and *Echidnophaga gallinacea*), as well as in ticks such as *Dermacentor nitens*, *Rhipicephalus microplus* (Table 1). Additionally, this *Rickettsia* has been detected in cat tissues in California, USA, suggesting pathogenicity for mammals. However, relying solely on molecular detection limits our understanding of its biology, physiology and actual pathogenic potential. Obtaining and maintaining a viable, pure isolate remains a major bottleneck in rickettsiology, yet it is essential for performing comprehensive phenotypic, ultrastructural and high-quality genomic characterizations.

**Fig. 1.**
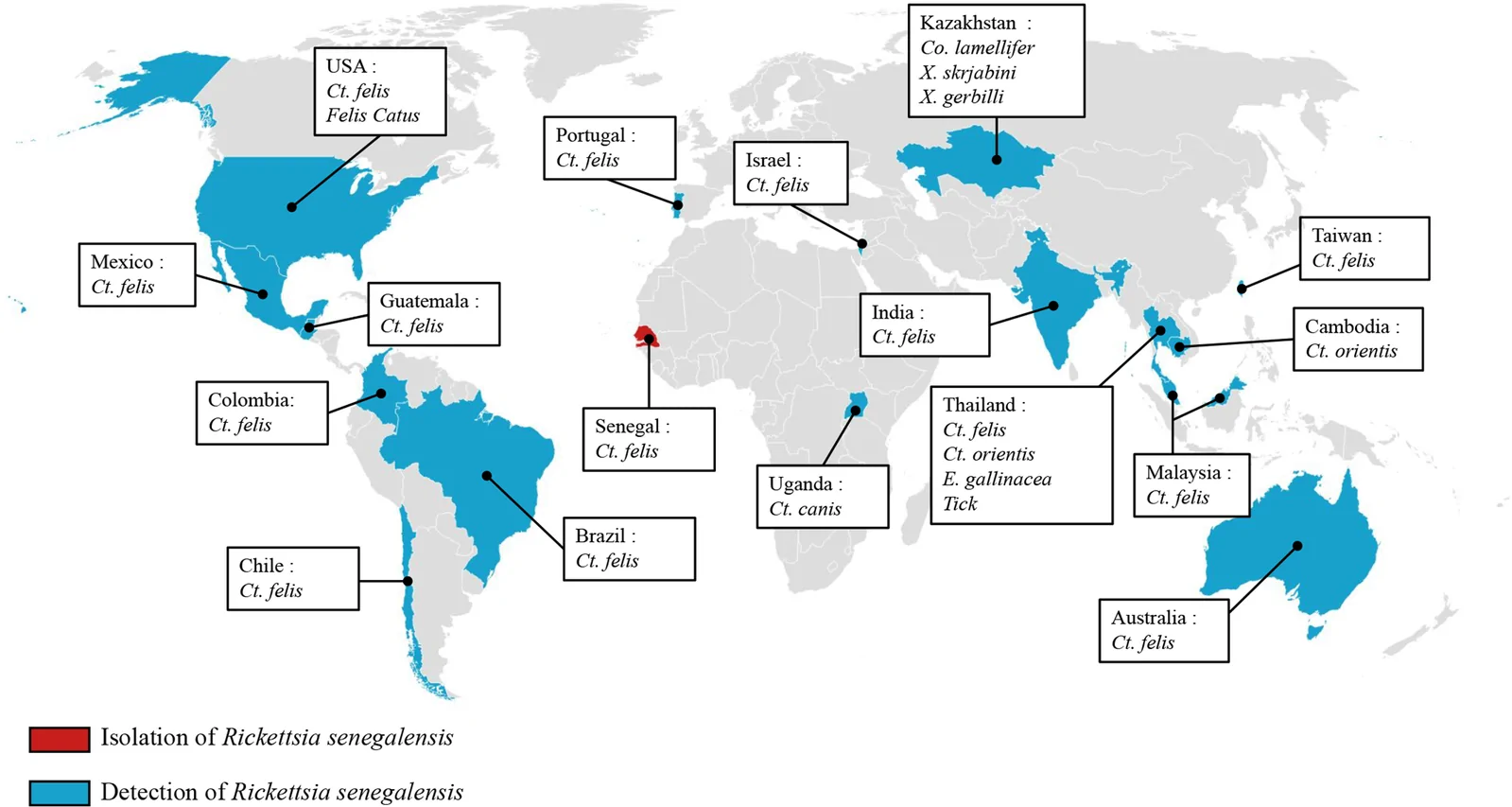
Global distribution of *R. senegalensis* and its hosts. Reported occurrences include fleas (*Ctenocephalides felis*, *Ctenocephalides canis*, *Ctenocephalides orientis*, *Coptopsylla lamellifer*, *X. skrjabini*, *X. gerbilli*, *E. gallinacea*), ticks and domestic cats (*Felis catus*) across 17 countries, with isolates obtained from fleas (*Ct. felis*) in Senegal.

**Table 1.** Summary of the identification of *R. senegalensis* reported to date. The table includes information on the country, designation, date, collection source, host, the prevalence of samples from fleas, ticks and cats testing positive for *R. senegalensis* and corresponding references

Country	Designation	Date	Collection source	Host	No. of samples tested and prevalence	Ref
Senegal	Strain PU01-02^T^	2012	Cat	*C. felis*	5 positive fleas/29 fleas	[21]
California, USA	Isolate Cf US 0036D	2012	Opossums	*C. felis*	3 positive fleas/597 fleas	[32]
Taiwan	Isolate HA0985	2012	Cat	*C. felis*	nd	GenBank (accession: MZ773249)
Brazil	Isolate 19P	2013	Opossums	*C. felis*	1 positive flea/54 fleas	[44]
Australia	Isolate AL357-5	2014	Cat	*C. felis*	1 positive flea/11 fleas	[45]
Kazakhstan	nd	2014	*Rhombomys opimus*	*Coptopsylla lamellifer*	374 fleas – 1 positive pool/40 pools	[33]
				*X. skrjabini*	1,444 fleas – 1 positive pool/73 pools	
				*X. gerbilli*	532 fleas – 6 positive pools/87 pools	
India	Isolate RAP2CF15	2015	Dog	*C. felis*	4 positive fleas/16 fleas	[46]
Marianna Islands, USA	nd	2015	*Rusa marianna*	*R. microplus*	1 positive tick/78 ticks	[47]
Oklahoma, USA	nd	2016	Privately-owned cat	*C. felis*	222 fleas – 1 positive pool/72 pools	[36]
Chile	nd	2016	Rural dogs (negative PCR)	*C. felis*	126 fleas – 1 positive pool/68 pools	[48]
Colombia	Isolate P227	2017	*Equus caballus*	*D. nitens*	2,809 ticks – 1 positive pool/602 pools	[49]
Cambodia	nd	2017	*Canis familiaris*	*C. orientis*	2 positive fleas/126 fleas	[50]
Israel	Isolate CtlS-18	2017	Cat	*C. felis*	2 positive fleas/91 fleas	[51]
Texas, USA	Isolate F112	2017	*Felis catus*	*C. felis*	721 fleas – 28 positive pools/140 pools	[52]
				*F. catus*	1 positive cat/24 cats	
Texas, USA	Isolate 21.1	2017	Cat	*C. felis*	314 fleas – 6 positive pools/87 pools	[53]
Uganda	Isolate MUWRP-0110	2017	Rabbit	*C. canis*	353 fleas – 1 positive pool/62 pools	[54]
California, USA	Isolate CA05Ctfe	2018	Cat	*C. felis*	77 fleas – 5 positive pools/64 pools	[38]
				*F. catus*	2 positive cats/30 cats	
Thailand	Isolate G14-447B	2018	Domestic animal	*C. felis*	88 fleas – 1 positive pool/12 pools	[37]
Colombia	nd	2019	Dog	*C. felis*	1,242 fleas – 1 positive pool/245 pools	[55]
Malaysia	Isolate DB 37	2019	Cat	*C. felis*	nd	[56]
Thailand	Isolate THAI	2020	Dog	*C. orientis*	1 positive flea/248 fleas	[34]
Thailand	nd	2021	Dog	*C. felis*	15 positive fleas/95 fleas	[35]
	nd	2021	Dog	*E. gallinacea*	1 positive flea/95 fleas	
	Isolat 706A	2021	Dog	Tick	1 positive tick/3 ticks	
Guatemala	nd	2022	Dog	*C. felis*	1 positive flea/433 fleas	[57]
India	nd	2022	Cat	*C. felis*	39 fleas – 1 positive pool/8 pools	[58]
			Dog	*C. canis*	18 fleas – 1 positive pool/6 pools	
Mexico	Isolate MTY	2023	Dog	*C. felis*	nd	GenBank (accession: OR651412)
India	Isolate G5	2023	Domestic animals and rodents	*C. felis*	90 fleas – 1 positive pool	[41]
Portugal	nd	2025	Cat	*C. felis*	1 positive flea/1,188 fleas	[59]

nd, No data.

To address this, we present the formal description of *R. senegalensis* sp. nov. based on the thorough characterization of its type strain, PU01-02ᵀ, which was successfully adapted to *in vitro* culture. This study provides the crucial biological and genomic foundation required to validly publish the name *R. senegalensis* and enable future experimental research on this emerging pathogen.

## Methods

### Origin and primary isolation of strain PU01-02^T^

*R. senegalensis* strain PU01-02ᵀ was originally isolated in 2012 from *C. felis* cat fleas collected from cats in Dakar, Senegal [21]. As previously described, live fleas were homogenized and inoculated into XTC-2 cell monolayers using the shell-vial centrifugation technique at 28 °C. Rickettsial infection was monitored over 2 weeks by Gimenez staining and confirmed by specific *gltA* quantitative PCR.

### Cell culture

Strain PU01-02^T^ of *R. senegalensis* was maintained in cell culture using monolayers of *Xenopus laevis* XTC-2 cells incubated at 28 °C in a CO_2_-free atmosphere. Rickettsial cultures were maintained in 25 cm² flasks with Leibovitz’s L-15 medium (Thermo Fisher Scientific, Illkirch-Graffenstaden, France), supplemented with 5% FBS (Thermo Fisher Scientific) and 8% tryptose phosphate broth (Sigma-Aldrich Chimie Saint-Quentin-Fallavier, France). Strain PU01-02^T^ was also inoculated into *Spodoptera frugiperda* SF9 cells and *Lymantria dispar* LD652 cells, incubated at 28 °C in TC-100 medium (Thermo Fisher Scientific) supplemented with 5% FBS. Additionally, the strain was cultured in transformed mouse (*Mus musculus*) fibroblast L929 cells, incubated at 37 °C with 5% CO2 in Dulbecco’s Modified Eagle’s Medium (DMEM, Thermo Fisher Scientific) supplemented with 5% FBS. Detection of rickettsiae was performed using Gimenez staining and quantitative PCR, following a previously described protocol and primers [12].

### Molecular analyses

Initially, the sequencing of key taxonomic markers (*gltA*, *ompB*, *rrs* and *sca4*) was performed for preliminary identification and compliance with standard rickettsial taxonomic guidelines, which was subsequently complemented and validated by whole-genome sequencing. DNA was extracted from the cell culture supernatant using the EZ1 automated system and the DNeasy Tissue Kit (Qiagen, Hilden, Germany). The *gltA* (1,250 bp), *ompB* (4,858 bp), *rrs* (1,421 bp) and *sca4* (1,677 bp) genes were sequenced using previously described primer pairs [22]. Amplicons were purified using Macherey–Nagel purification plates (Macherey–Nagel EURL, Hoerdt, France) and sequenced with the BigDye Terminator Cycle Sequencing Kit (Thermo Fisher Scientific). Sequencing reactions were analysed on an Applied Biosystems 3500 Genetic Analyzer (Thermo Fisher Scientific). The resulting sequences were assembled using ChromasPro software (version 1.7; Technelysium Pty Ltd., Tewantin, Australia). Multiple sequence alignments were performed with muscle implemented in mega X (Molecular Evolutionary Genetics Analysis version X), and phylogenetic trees based on the *gltA* and *ompB* genes were inferred using the maximum likelihood method in mega X, applying the Kimura two-parameter model with 1,000 bootstrap replicates.

### Genomic sequencing

Following assembly, host-derived (XTC-2) contigs were bioinformatically filtered out and removed based on sequence coverage and blastn searches against the host database. XTC-2 cells and bacteria from five 150 cm² flasks were collected and purified following a previously established protocol [23]. After a 2 h enzymatic lysis with proteinase K at 56 °C, genomic DNA was extracted using the EZ1 DNA Tissue Kit on the EZ1 automated system (Qiagen, Hilden, Germany). DNA quantification was performed using the Qubit High-Sensitivity Assay (Thermo Fisher Scientific). Genomic DNA was sequenced using MiSeq technology (Illumina Inc., San Diego, CA, USA) and the GridION platform (Oxford Nanopore Technologies, Oxford, UK), as previously described [24].

Reads obtained from Illumina MiSeq and Nanopore sequencing were assembled using Unicycler [25]. Following assembly, host-derived (XTC-2) contigs were bioinformatically filtered out and removed based on sequence coverage and blastn searches against the host database. OrthoANI (Orthologous Average Nucleotide Identity) was used to evaluate the genomic similarity between strain PU01-02^T^ and the species with validly published names of the TRG, applying a species delineation threshold of 99.19% [26]. Furthermore, the Genome-to-Genome Distance Calculator 2.1 web server (https://tygs.dsmz.de/) was utilized to determine the digital DNA–DNA hybridization (dDDH) values between the selected genomes. A threshold of less than 92.30% using formula 2 was applied to define species boundaries, and a genomic tree was constructed with Type Strain Genome Server (TYGS) [27].

### Electron microscopy

For ultrastructural observation, infected XTC-2 cells underwent fixation using 2.5% glutaraldehyde in a 0.1 M sodium cacodylate buffer at 4 °C for 5 h. Resin embedding was executed with a PELCO BiowavePro+ (Ted Pella, Redding, USA) microwave device. The specimens were then sequentially dehydrated through an increasing gradient of ethanol (50%, 70% and 96%) prior to infiltration with medium-grade LR-White resin (Polysciences, Warrington, USA). After a 72 h polymerization phase at 60 °C, 100 nm ultrathin sections were prepared with a Leica UC7 ultramicrotome and recovered onto HR25 300-mesh copper/rhodium grids (TAAB, Aldermaston, UK). Post-staining of the sections was performed using uranyl acetate and lead citrate for contrast enhancement. Finally, grids were fixed onto a glass slide with double-sided adhesive tape and coated with platinum. Specimen grids were observed by scanning electron microscopy (SEM) using a SU5000 microscope (Hitachi High-Technologies, Tokyo, Japan) operated under high vacuum at an accelerating voltage of 7 kV, in observation mode with a spot size of 30 and a backscattered electron (BSE) detector, following a previously described protocol [28].

## Results

### Primary isolation

Primary isolation from *C. felis* fleas collected in Dakar, Senegal yielded three rickettsial strains (PU01-02, PUX1-X2 and PU03-04) in XTC-2 cells [21]. All exhibited the typical intracellular Gimenez-positive morphology. Among these isolates, strain PU01-02ᵀ was selected for the present taxonomic characterization.

### Cell culture

*R. senegalensis* was maintained on XTC-2 cell monolayers for 29 passages. The SF9 and LD652 cell lines also supported satisfactory growth of the strain PU01-02^T^ (Fig. 2). After 28 days, the cycle threshold (Ct) values obtained for these three cell types were 17.05, 17.98 and 19.76 for XTC-2, SF9 and LD652 cells, respectively. In all three cell lines (XTC-2, SF9 and LD652), rickettsiae were observed both intra- and extracellularly during continuous culture, with no cytopathic effects. In contrast, no detection of the bacterium was achieved by Gimenez staining or quantitative PCR in L929 cells after 40 days post-inoculation.

**Fig. 2.**
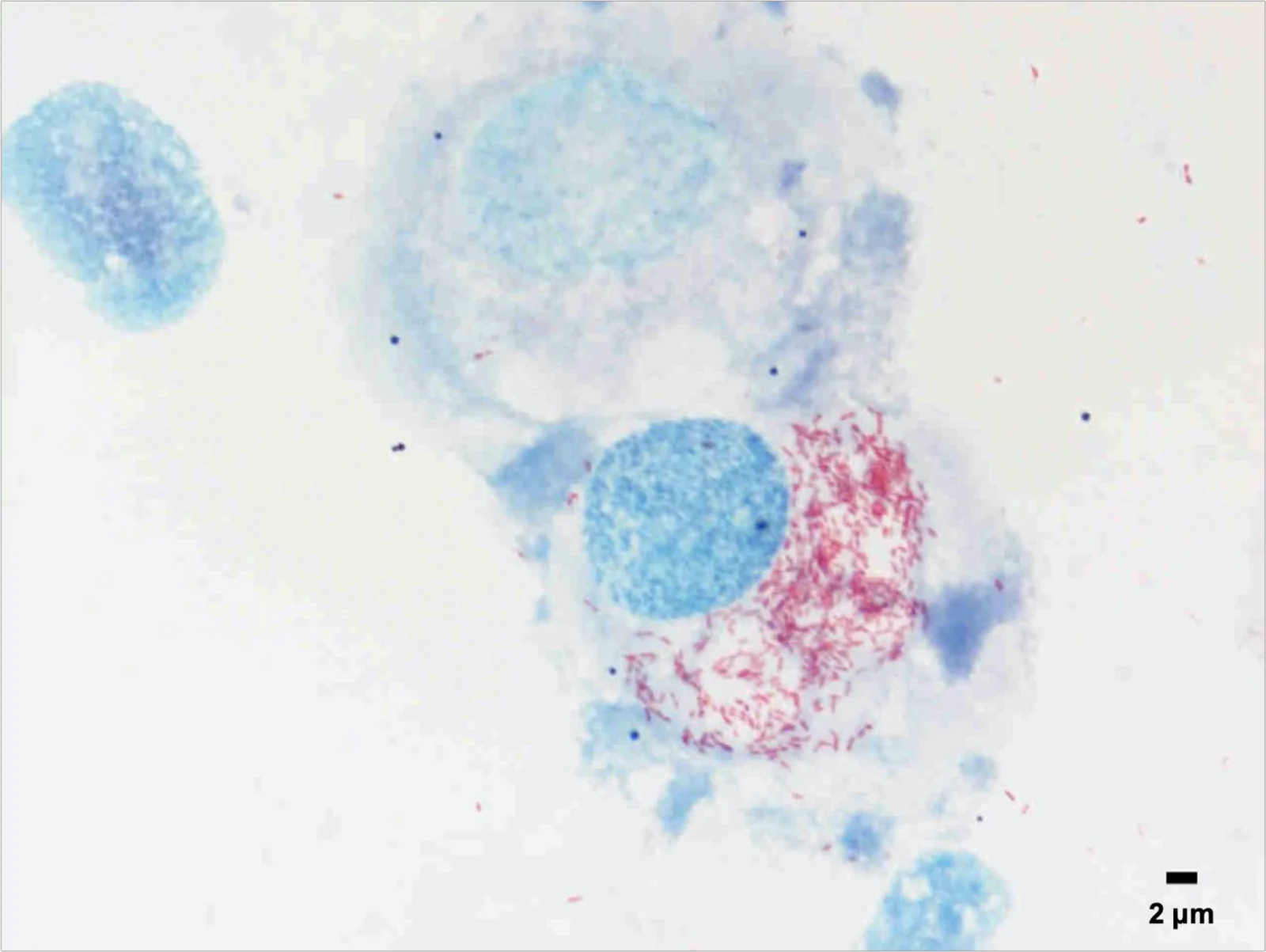
*S. frugiperda* (*Lepidoptera*) SF9 cell line infected with *R. senegalensis* sp. nov. strain PU01-02^T^, Gimenez staining, 1500×.

### Molecular identity analyses

blast sequence comparison for the *gltA* gene revealed that the strain PU01-02^T^ shares 100% similarity with *R. senegalensis* strain RAPCF15, which was identified in *C. felis* fleas collected in India. Additionally, a 99.91% similarity (1,149/1,150 bp) was found with *Rickettsia* sp. RF31, an isolate obtained from *C. canis* fleas collected from a dog in Thailand. The closest species with validly published names, *R. asembonensis* 8556D1, showed 98% (1,105/1,127 bp) similarity. For the *ompB* gene, a 99.81% (4,285/4,293 bp) sequence similarity was observed with *Candidatus* R. senegalensis Cf_US_0036D (identified in *C. felis* fleas collected from opossums in the USA), while the highest similarity among species with validly published names was found with *Rickettsia rhipicephali* strain HJ#5 (91.08%, 4,440/4,875 bp). Regarding the *sca4* gene, the blast results for the strain PU01-02^T^ demonstrated 100% (1,050/1,050 bp) and 97.17% (1,612/1,659 bp) sequence similarity with the *R. senegalensis* Cf_US_0036D and *R. felis* clone As3, respectively. These comparisons also support the genetic cohesion of *R. senegalensis*, as PU01-02^T^ showed complete sequence identity with previously reported *R. senegalensis* strains for both *gltA* and *sca4*, whereas sequence divergence was observed from the closest species with validly published names. In particular, the *gltA* and *sca4* similarities with *R. asembonensis* and *R. felis*, respectively, indicate that PU01-02^T^ can be discriminated from these closely related species at the molecular level. As the sequence similarity values for all three genes fall below the established thresholds for defining a *Rickettsia* species, these results support the conclusion that strain PU01-02^T^ represents a new species [29].

### Phylogenetic analyses

Sequencing of the *gltA* and *ompB* genes enabled the phylogenetic analysis of *R. senegalensis* (Fig. 3). The phylogenetic trees showed that the strain PU01-02^T^, along with the other isolates of *R. senegalensis*, is positioned within the TRG.

**Fig. 3.**
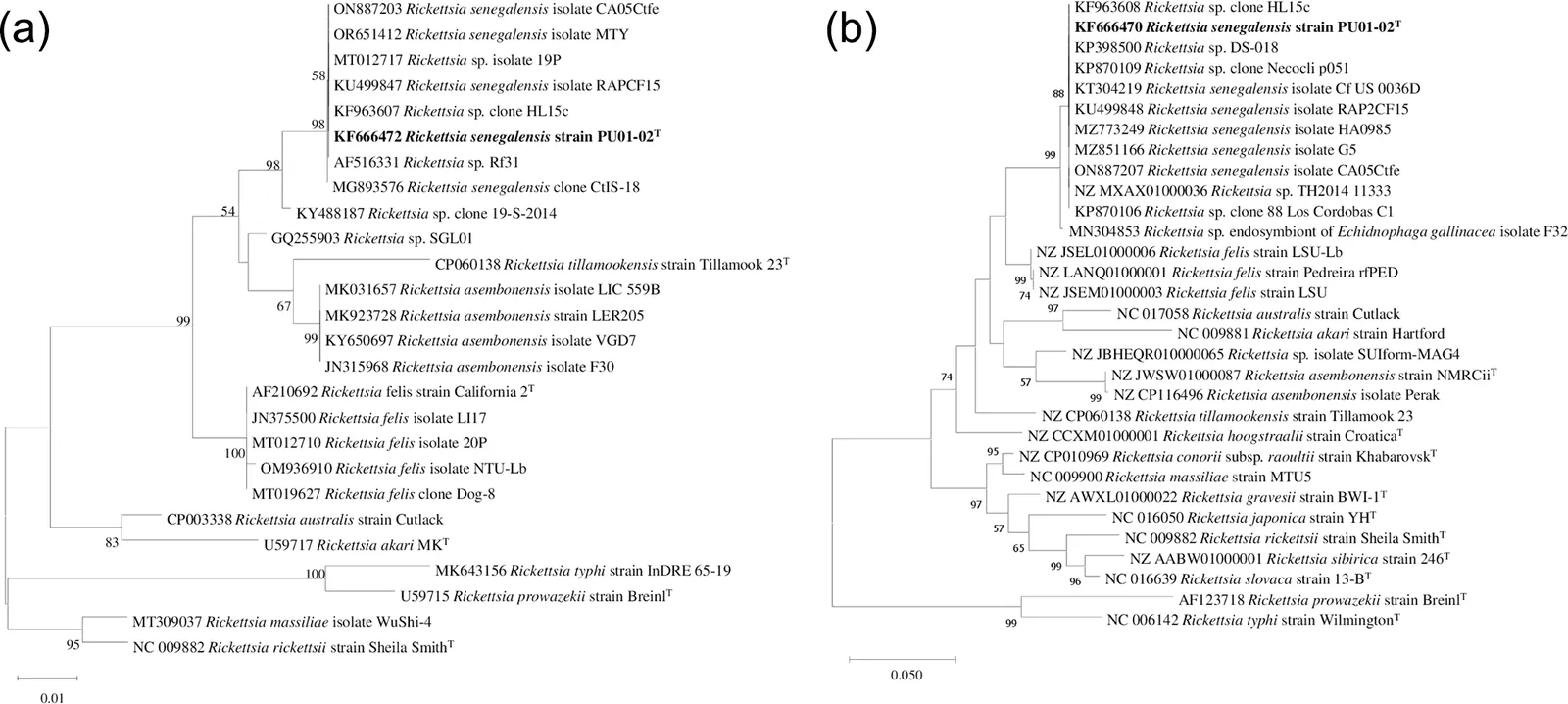
Phylogenetic trees of the *gltA* (**a**) and *ompB* (**b**) genes for *R. senegalensis* strain PU01-02^T^ and its closest species. Phylogenetic trees were reconstructed using the maximum likelihood method under the Kimura two-parameter model, with branch support assessed by 1,000 bootstrap replicates. Total length 1,250 bp for *gltA* and 4,858 bp for *ompB*.

### Genomic analyses

Fig. 4a displays a genomic tree of the strain PU01-02^T^. This strain harbours a circular chromosome with an estimated size of 1,626,546 bp and a G+C content of 33.2%, consistent with previously reported values for other rickettsiae [30]. Genome annotation identified 1,986 protein-coding genes, along with 33 tRNA genes and 3 rRNA operons. The strain PU01-02^T^ shows the highest dDDH value with *R. felis* strain Pedreira, reaching 65.30%, while the maximum OrthoANI value is 95.95% with the same strain (Table S1, Fig. 4b). Both values fall below the established species delineation thresholds, confirming that *R. senegalensis* represents a novel species [31]. The fact that *R. felis* strain Pedreira represents the closest genome based on both dDDH and OrthoANI is particularly relevant for species discrimination. Despite this close genomic relationship, the 95.95% OrthoANI and 65.30% dDDH values remain below the accepted thresholds for species discrimination, indicating that PU01-02^T^ does not belong to the *R. felis* genomic species. Thus, the genome-wide data provide a stronger discriminatory criterion than individual marker genes alone and establish *R. senegalensis* as a distinct genomic lineage within the TRG.

**Fig. 4.**
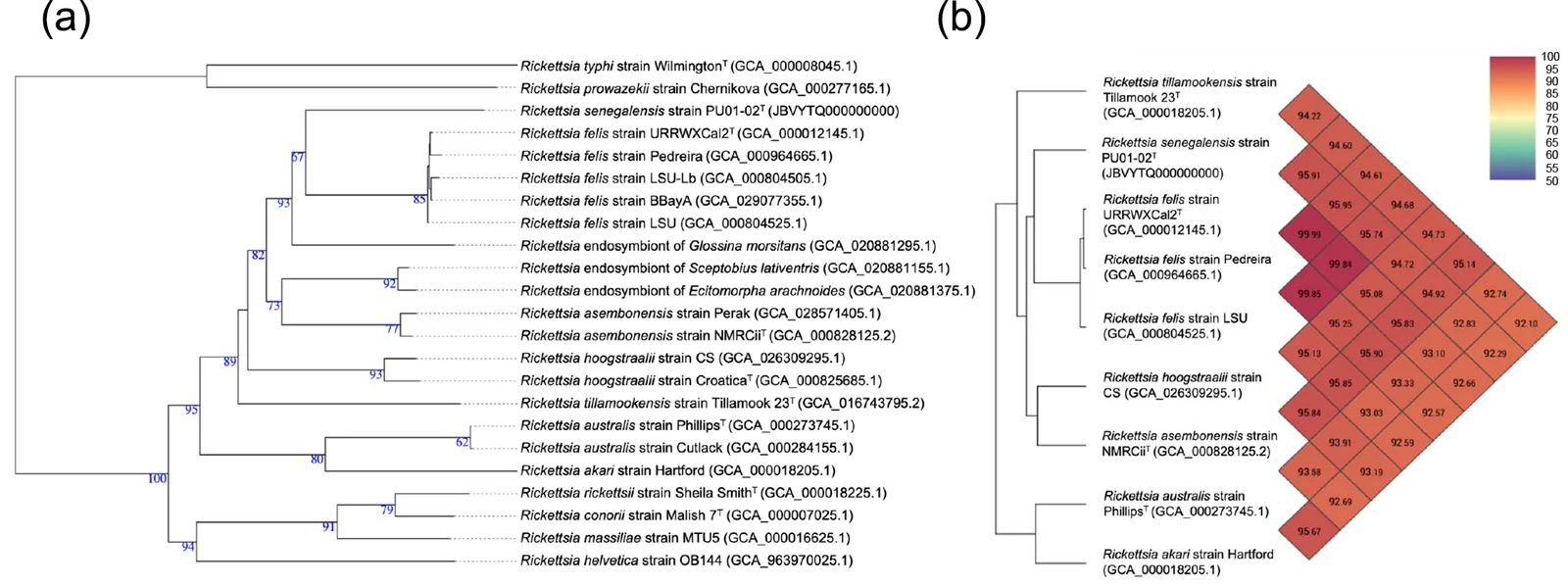
(**a**) Genomic tree of *R. senegalensis* strain PU01-02^T^ and closely related species constructed using TYGS. (**b**) OrthoANI values calculated using genome sequences of *R. senegalensis* strain PU01-02^T^ and TRG *Rickettsia* species with validly published names.

Additionally, three plasmid contigs, designated pRS1, pRS2 and pRS3, were detected through next-generation sequencing (NGS) sequencing, with respective sizes of 59,188, 28,830 and 50,788 bp. The plasmid pRS1 appeared to be unique, sharing only 88.03% nt identity over 63% coverage with the pRra2 plasmid from *Rickettsia raoultii* strain Khabarovsk. The highest similarity observed for plasmid pRS2 was 93.27%, with a limited coverage of 22%, when compared to the genome assembly of the *Rickettsia* endosymbiont of *Cantharis rufa* isolate 54716. Finally, plasmid pRS3 exhibited a maximum identity of 94.57% (42% coverage) with the genome assembly of the *Rickettsia* endosymbiont of *Pantillius tunicatus* isolate 54737, and 92.51% identity with 28% coverage when compared to the pRF plasmid of *R. felis*. Altogether, this distinct plasmid profile provides an additional genomic feature distinguishing strain PU01-02ᵀ from *R. felis* and other related rickettsiae.

### Electron microscopy

The microscopic characterization of strain PU01-02^T^ was performed by SEM of infected cell cultures (Fig. 5). Multiple bacteria exhibited a morphology typical of other *Rickettsia* species, appearing as rod-shaped cells ~1.5 µm in length and 0.3 µm in width. The bacteria were located exclusively within the host-cell cytoplasm, where they occurred either individually or in dense clusters, without evidence of vacuolar sequestration, consistent with active intracellular replication. The host-cell nucleus and cytoplasmic boundaries remained clearly distinguishable, while the bacteria were distributed throughout the cytoplasm surrounding the nucleus.

**Fig. 5.**
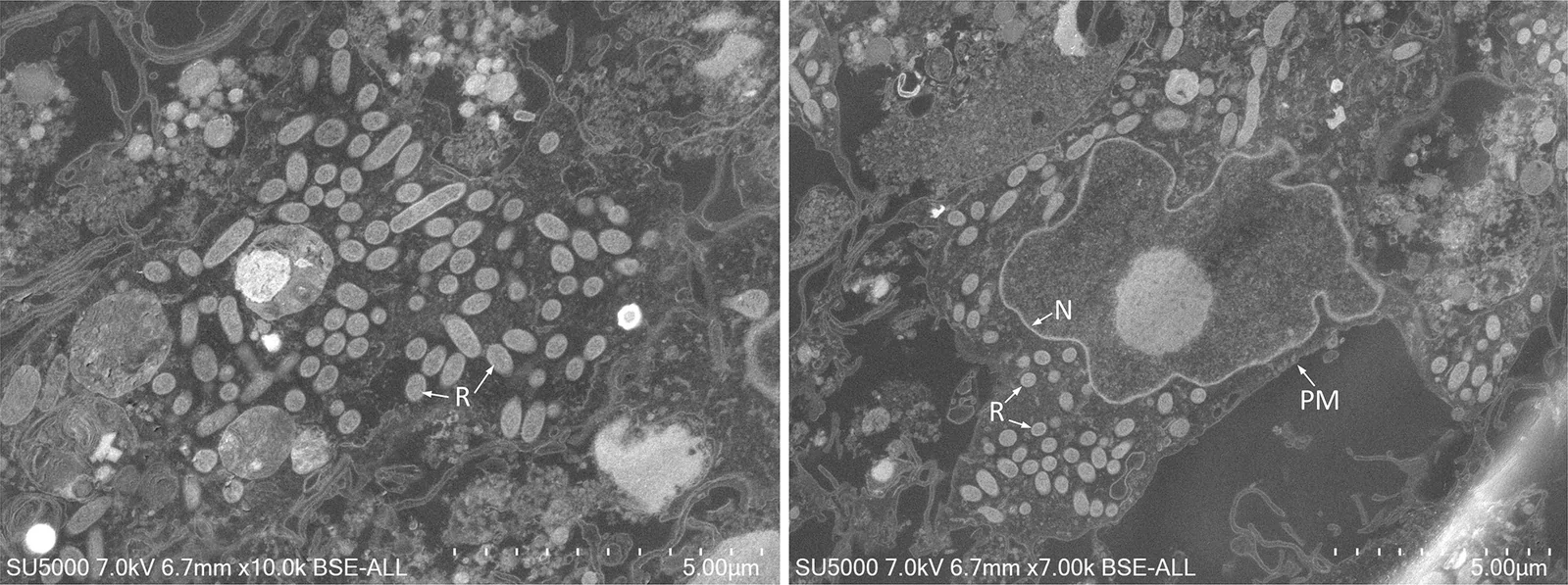
SEM and BSE mode images of *R. senegalensis* strain PU01-02^T^ infecting XTC-2 cells. R, *R. senegalensis* cells; N, host-cell nucleus; PM, plasma membrane. Scale bars=5 µm.

## Discussion

In this study, we formally describe *R. senegalensis* sp. nov., a novel *Rickettsia* species isolated in 2012 from *C. felis* cat fleas collected in Dakar, Senegal [21]. Prior to this formal taxonomic description, this bacterium was provisionally referred to as *Candidatus* R. senegalensis in the literature.

The successful isolation, stable *in vitro* cultivation and polyphasic characterization of *R. senegalensis* strain PU01-02ᵀ represent a major milestone. Relying solely on molecular detection restricts our understanding to DNA presence, leaving the physiological and functional traits of the living bacterium unexplored. Obtaining a viable, pure isolate bypasses these limitations. Phenotypically, it allowed us to establish its unique optimal growth parameters (28 °C in insect and amphibian cell lines) and ultrastructural features, demonstrating its free cytoplasmic localization without vacuolar sequestration. Genomically, having a high-quality, non-metagenomic assembly allowed for the precise detection of three distinct plasmids. Plasmids are key drivers of horizontal gene transfer and evolution within the *R. felis* cluster; thus, the viability of this strain opens the door for future experimental studies to investigate plasmid-mediated virulence, replication kinetics and host-cell interactions under controlled laboratory conditions.

To clearly distinguish *R. senegalensis* sp. nov. from its closest relatives with validly published names within the TRG, particularly *R. felis* and *R. asembonensis*, genomic, molecular and phenotypic criteria were considered. The highest OrthoANI (95.95%) and dDDH (65.30%) values were obtained with *R. felis* but remained below the species delineation thresholds, supporting the separation of the two species. At the molecular level, strain PU01-02^T^ showed sequence divergence in *gltA*, *ompB* and *sca4* from species with validly published names, while sharing 100% identity for *gltA* and *sca4* with previously reported *R. senegalensis* strains. In addition, PU01-02^T^ possesses a distinct plasmid complement (pRS1, pRS2 and pRS3) compared with the pRF plasmid of *R. felis*. Together with its distinct phylogenetic position and its growth in XTC-2, SF9 and LD652 cells at 28 °C (coupled with the absence of growth in mammalian L929 cells at 37 °C), these features provide complementary evidence supporting the distinction of *R. senegalensis* from closely related TRG species.

Since its first detection, this species has been identified, across all continents except Antarctica, in various haematophagous arthropods, including four flea genera (*Ctenocephalides, Coptopsylla, Xenopsylla* and *Echidnophaga*), as well as hard tick species [32–35]. Some of these arthropods were collected from domestic animals, raising concerns about potential transmission to humans [36, 37]. Although the pathogenicity of *R. senegalensis* remains unknown, its close phylogenetic relationship with *R. felis* and *R. asembonensis* (both being responsible for vector-borne diseases) suggests a possible zoonotic risk. This concern is further reinforced by the detection of *R. senegalensis* DNA in the tissues of two unowned cats captured in the Americas [38]. Previous studies have also reported *Rickettsia* DNA in the blood of dogs and cats, although it remains unclear whether the infection is sufficiently prolonged and stable to establish host competence [39, 40]. Nonetheless, these mammals play a crucial role as primary hosts for fleas, facilitating bacterial amplification through horizontal transmission.

Moreover, *R. senegalensis* has been detected in fleas collected from rodents in both the Americas and India [32, 41]. This finding underscores the need for further investigation into the role of rodents in the epidemiological cycle of TRG species. This issue is particularly relevant as rodents, the most diverse and abundant mammalian order, are already well known as both carriers and reservoirs of numerous human pathogens [42, 43]. The major host and vector appears to be *C. felis*, a flea species with a global distribution. However, most detections of *R. senegalensis* have been reported in tropical and subtropical regions.

These findings highlight the importance of enhancing surveillance efforts not only on haematophagous arthropods but also on domestic animals, due to the high zoonotic risk associated with their close contact with humans. Additionally, monitoring wild animals is crucial to gaining a better understanding of the epidemiology of TRG rickettsiae. Although cat and dog fleas, *C. felis* and *C. canis*, appear to be the primary arthropod hosts, the ecology and distribution of these bacteria remain largely unknown [9]. Furthermore, *R. senegalensis* appears to be sympatric with other TRG species, such as *R. felis* and *R. asembonensis* [32]. Finally, formally describing *R. senegalensis* sp. nov. and depositing its type strain PU01-02ᵀ (=DSM 28250ᵀ =CSUR R184ᵀ) allows the valid publication of its name, transitioning it from a provisional *Candidatus* status. Making this fully characterized, viable reference strain publicly available is essential for the scientific community. It provides researchers with the physical material needed to perform comparative studies, avoid diagnostic misidentification within the complex *R. felis* cluster, and accurately track the global distribution and clinical relevance of this emerging *Rickettsia*.

## Description of *Rickettsia senegalensis* sp. nov

*Rickettsia senegalensis* (se.ne.ga.len′sis. N.L. fem. adj. *senegalensis*, from Senegal, the country in western Africa where the *Ctenocephalides felis* fleas were collected) is an obligate intracellular, Gram-negative bacterium that proliferates in *Xenopus laevis* XTC-2, *Spodoptera frugiperda* SF9 and *Lymantria dispar* LD652 cell lines at 28 °C in a CO_₂_-free atmosphere. The bacterium does not grow at 37 °C in *Mus musculus* L929 fibroblast cultures. The species can be differentiated from its closest phylogenetic relatives with validly published names (*Rickettsia felis* and *Rickettsia asembonensis*) by dDDH (65.30%) and OrthoANI (95.95%) values below the species threshold, distinct plasmid content (pRS1, pRS2 and pRS3) and sequence divergence in *gltA*, *ompB* and *sca4* genes below the established cutoff values for the genus *Rickettsia*. Phylogenetic analysis based on *gltA*, *ompB* and *sca4* gene sequences demonstrates that *R. senegalensis* is distinct from other *Rickettsia* species with validly published names. The PU01-02^T^ strain clusters with other *R. senegalensis* strains identified in various flea species. The genome size is 1.62 Mb, with a G+C content of 33.2%. The genome accession number is JBVYTQ000000000, and the plasmid accession numbers are PZ272915, PZ272916 and PZ272917 for pRS1, pRS2 and pRS3, respectively. The sequences corresponding to the *rrs*, *gltA*, *ompB* and *sca4* genes of the PU01-02^T^ strain have been deposited in GenBank under the accession numbers KF666476, KF666472, KF666470 and KF666474, respectively.

The type strain, PU01-02ᵀ (= DSM 28250ᵀ = CSUR R184ᵀ), was isolated from a *C. felis* flea collected in Dakar, Senegal, in 2012.

## Supplementary material

10.1099/ijsem.0.007294 Table S1.

## Abbreviations

BSE: backscattered electron
dDDH: digital DNA–DNA hybridization
DMEM: Dulbecco’s Modified Eagle’s Medium
OrthoANI: Orthologous Average Nucleotide Identity
SEM: scanning electron microscopy
TRG: transitionnal *Rickettsia* group
